# Substance Use and Psychological Distress in Mexican Adults during COVID-19 Pandemic: A Cross-Sectional Study

**DOI:** 10.3390/ijerph20010716

**Published:** 2022-12-30

**Authors:** Juan Carlos Ibarrola-Peña, Tania Abigail Cueto-Valadez, Jonathan Matías Chejfec-Ciociano, Luis Rodrigo Cifuentes-Andrade, Andrea Estefanía Cueto-Valadez, Guadalupe Castillo-Cardiel, Guillermo Alonso Cervantes-Cardona, Enrique Cervantes-Pérez, Gabino Cervantes-Guevara, Mario Jesús Guzmán-Ruvalcaba, José Héctor Sapién-Fernández, José Aldo Guzmán-Barba, Isaac Esparza-Estrada, Paola Flores-Becerril, Irma Valeria Brancaccio-Pérez, Bertha Georgina Guzmán-Ramírez, Andrea Socorro Álvarez-Villaseñor, Francisco José Barbosa-Camacho, Emilio Alberto Reyes-Elizalde, Clotilde Fuentes-Orozco, Alejandro González-Ojeda

**Affiliations:** 1 Hospital General y Medicina Familiar de Zona No. 2, Instituto Mexicano del Seguro Social, Monterrey 64010, Nuevo León, Mexico; 2 Unidad de Investigación Biomédica 02, Hospital de Especialidades del Centro Médico Nacional de Occidente, Instituto Mexicano del Seguro Social, Guadalajara 44340, Jalisco, Mexico; 3 Departamento de Disciplinas Filosófico, Metodológicas e Instrumentales, Centro Universitario de Ciencias de la Salud, Universidad de Guadalajara, Guadalajara 44340, Jalisco, Mexico; 4 Departamento de Medicina Interna, Hospital Civil de Guadalajara Fray Antonio Alcalde, Centro Universitario de Ciencias de la Salud, Universidad de Guadalajara, Guadalajara 44340, Jalisco, Mexico; 5 Departamento de Bienestar y Desarrollo Sustentable, Centro Universitario del Norte, Universidad de Guadalajara, Colotlán 46200, Jalisco, Mexico; 6 Departamento de Pediatría, Hospital Civil de Guadalajara Fray Antonio Alcalde, Universidad de Guadalajara, Guadalajara 44340, Jalisco, Mexico; 7 Instituto Mexicano del Seguro Social, La Paz 23060, Baja California, Mexico; 8 Departamento de Traumatología y Ortopedia, Instituto Nacional de Rehabilitación Luis Guillermo Ibarra Ibarra, Tlalpan, Ciudad de Mexico 14389, Mexico

**Keywords:** addiction, anxiety, assessment, depression, drug and alcohol use, psychometrics

## Abstract

Objectives: This observational cross-sectional study examined changes in substance use during the coronavirus disease 2019 (COVID-19) pandemic in the Mexican population and evaluated whether depression or anxiety was associated with these new consumption patterns. Methods: An online survey was distributed to the general population. Participants were questioned about their demographics, situation during the COVID-19 pandemic, and substance consumption patterns. The Patient Health Questionnaire-9 for depression and the Coronavirus Anxiety Scale were used. Results: A total of 866 individuals completed the survey. The mean scores for the Patient Health Questionnaire-9 and Coronavirus Anxiety Scale were 8.89 ± 6.20 and 3.48 ± 3.22, respectively. The preferred substances were alcohol (19%), tobacco (16.5%), and marijuana (5.6%). Consumption of alcohol (*p* = 0.042) significantly increased during the pandemic and it was higher in women than in men (*p* = 0.040). Conclusions: Substance use patterns were affected by the pandemic, with an increase in the number of users and consumption rate, as well as the reported psychiatric symptoms.

## 1. Introduction

Since late 2019, the world has faced the coronavirus disease 2019 (COVID-19) pandemic, which has brought about profound societal changes. The measures taken by governments to reduce exposure to viral particles, such as the closure of public places, home isolation, and social distancing, are also factors associated with psychological disturbances, such as depression, stress, anger, and confusion [1]. Loneliness appears to predispose individuals to depression, social anxiety, generalized anxiety, and suicidal ideation [2]. Due to the effects of the pandemic on daily life, the United Nations and the World Health Organization described it as a social, economic, health, and human crisis [2,3]. Although a pandemic affects the general population, such conditions primarily affect people in vulnerable social groups, such as the elderly, impoverished people, and children [4]. A common social response to life stressors is the use of one or more substances, such as tobacco, alcohol, or over-the-counter medications [5]. However, the use of such substances has been related to cognitive alterations such as memory, attention, speech, and executive function problems [6]. Aside from the physical alterations, this can negatively affect all aspects of the user’s life, including family relationships and the workplace [7]. As anticipated, the newly discovered SARS-CoV-2 infection brought with it several stressors, such as the uncertainty of the virus’s clinical evolution, the absence of a treatment or vaccine, and the economic strain of the mandatory lockdown, which led to an increase in overall stress and, consequently, substance and drug abuse [8,9]

During this pandemic, individuals who already used drugs were left vulnerable due to the prioritization of patients with COVID-19 in the healthcare systems. In addition, physically attending cognitive behavioral therapy or visiting a healthcare facility to receive pharmacological treatment could increase the risk of infection [10,11,12].

Nowadays, the literature reports the outcomes of international investigations employing comparable methodologies. In May of 2020, a study conducted in the United States revealed a correlation between the COVID-19 pandemic and the population’s response in alcoholic beverage usage, concluding that monitoring the impact of the pandemic on this pertinent public health concern is crucial [13]. Using a self-perception questionnaire on changes in drug use, a study of 36,538 adults from 21 European countries was carried out during the midpoint of the year 2020. The study indicated rising trends in the use of tobacco and cannabis, but a decline in alcohol consumption [14]. A cross-sectional study conducted in November 2020 in the United Kingdom found a significant increase in alcohol consumption during the pandemic among older, essential workers, individuals with children, those with a personal relationship with a severely ill COVID-19 patient, and those with higher levels of depression and anxiety [15]. In June 2021, a study of 1065 people in Greece revealed that during the pandemic, 43.7% of the population lowered their alcohol use and 67.4% of frequent cannabis users abstained. In the case of tobacco, one in three smokers reportedly acknowledged smoking more during the COVID-19 outbreak [16].

The present study aimed to identify the changes in substance and drug use before and during the COVID-19 pandemic as well as evaluate whether depression or anxiety affected the consumption pattern in the Mexican population.

## 2. Materials and Methods

### 2.1. Design

The study design was a cross-section study in which participants answered an online anonymous survey between April 2021 and June 2021. The inclusion criteria for the study were as follows: adults aged 18 years or older; and individuals who completed the survey and provided acknowledgment and approval through informed consent. The exclusion criterion was individuals with an incomplete survey. The sample size was determined using the Kelsey et al. formula, in which the difference in substance use proportions was calculated.

$$\frac{{\left(Z\alpha +Z\beta \right)}^{2}*p\left(1-p\right)\left(r+1\right)}{r{\left(p0-p1\right)}^{2}}$$

It was calculated according to the statistics provided in the Substance Abuse and Mental Health Services Administration Annual National Report by the U.S. Department of Health and Human Services [17], which gives a 14.8% prevalence rate of substance use, compared with the 10.3% prevalence rate noted in the Mexican national survey report on drug use [18]. A minimum sample size of 836 individuals was calculated using an error of 0.05 and b error of 0.10. The target demographic was the general populace. The survey was distributed online using the Google Forms (Google, Mountain View, CA, USA) platform. Using a snowball sampling technique, participants were asked to distribute the questionnaire to their acquaintance. A link to the survey was shared and circulated via social networks and email, along with information on the significance of participating in the study and an email to answer the participants’ concerns. By signing an informed consent form, each participant was informed and gave consent to participate in the study. If a user wanted to amend an answer, he could return to earlier questions. The questionnaire was completed in complete anonymity. The survey data were exported to a database by an automated method, which was then examined by the research team.

### 2.2. Instruments

Twenty individuals completed a pilot questionnaire to evaluate the difficulty and comprehension of the questions. The final questionnaire has five sections and a total of 85 multiple-choice and five open-ended questions. After the participants had read and accepted the informed consent guidelines, they were invited to answer the survey, in which we used three instruments. Demographic data were sought to evaluate the sample. The primary survey was based on the instrument used by MacMillan et al. [19]. The main topics addressed in the questionnaire included: the sources used to find information about COVID-19 and their credibility; if the respondent or a family member had been infected; difficulties regarding social, economic, and emotional limitations during the pandemic; assistance from religious or support groups; and the use of substances to relieve stress before and during the pandemic. Use of substances was measured as follows: On a list of substances, consumption was asked as a dichotomous variable. The individual then indicated the amount of consumption prior to the pandemic and after the pandemic began. The quantity was measured using standard units, such as 355 mL of alcohol per day and the number of cigarettes. The following items are examples of questionnaire items: Did your religious or support community offer you emotional or other forms of assistance? During the pandemic, did you use substances to cope with the pressure and stress? How many glasses of alcohol (355 mL) did you consume prior to the pandemic? How many glasses (355 mL) of alcohol do you consume on a daily basis during the pandemic? Did you incur debt due to the pandemic? Since the objective was to evaluate the increase or decrease in consumption, we left it up to the user to determine the average size of their portion consumed.

The prevalence of depression was assessed using the Spanish version of the Patient Health Questionnaire-9 (PHQ-9) [20]. This is a nine-question scale using the Likert scale scores ranging from 0 to 3, in which the participant must select the answer that best represents their mood in the previous 2 weeks. The total score is divided into several cut points; overall, a score >10 suggests the presence of depression.

In addition, the Spanish validated version of the Coronavirus Anxiety Scale (CAS) was used to assess the participants’ anxiety and stress responses during the COVID-19 pandemic [21,22]. Similar to the PHQ-9, this is a five-item questionnaire using the Likert scale scores ranging from 0 to 4. The participants must select the answer that best represents their feelings toward the COVID-19 pandemic. Participants with a score 39 are classified as having dysfunctional anxiety.

### 2.3. Data Analysis

Statistical analysis was performed using IBM SPSS Statistics for Windows, version 25.0 (IBM Corp, Armonk, NY, USA). A descriptive study was conducted using measures of central tendency. For inferential analysis, the chi-square and Mann–Whitney-*U* tests were performed depending on the data distribution and following one-way analysis of variance. For quantitative variables, paired *t*, Student’s *t*, McNemar or Wilcoxon tests were performed depending on the data distribution. The change in dose and substance use was analyzed using a percentage change. Results with a *p*-value < 0.05 were considered statistically significant.

## 3. Results

**Figure 1:**
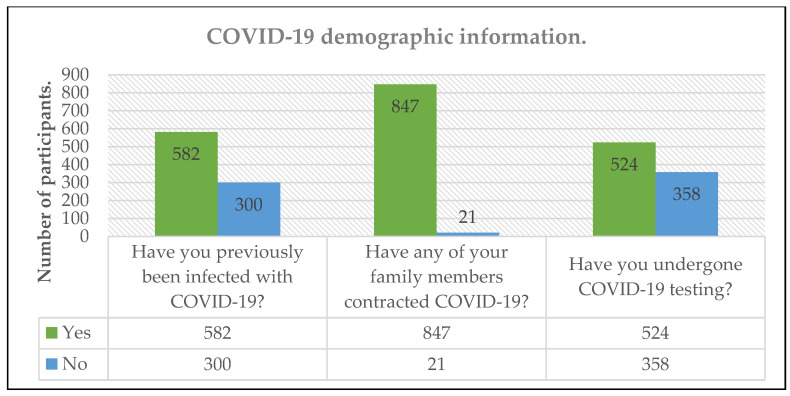
Demographic characteristics of the study.

A total of 880 individuals answered the survey. Due to the sampling method, there is no information regarding the number of individuals that got the survey; hence, there is no participation rate. We had a 98.4% completion rate, with 14 respondents not answering the complete questionnaire. Incomplete questionnaires were excluded during the analysis. The demographic characteristics of the 866 participants included are presented in Table 1 and Figure 1.

### 3.1. Substance Use

Among the 866 participants, 196 (22.5%) reported using a substance to deal with stress. The most prevalent substance consumed was alcohol (19%), followed by tobacco (16.5%), and marijuana (5.6%). Other drugs, such as cocaine and hallucinogenic mushrooms, represented only 0.2% of the sample. Regarding participants’ consumption habits before and during the pandemic, a significant increase in the use of alcohol (*p* = 0.006), and tobacco (*p* = 0.001) was observed. On analyzing the number of consumers by substance before and after the pandemic, an increment in the number was noted for alcohol (4.2%), and marijuana (2%). However, there was a decrease in the number of tobacco users (11.1%), which was statistically significant (*p* = 0.001). A detailed description of substance use is presented in Table 2.

### 3.2. Psychological Impact

The prevalence of all psychiatric pathologies and the questionnaire scores are presented in Table 3. The most prevalent baseline psychiatric diagnosis found in the sample was anxiety (95 [10.9%]) and depression (78 [8.7%]). In the PHQ-9 test performed to screen for depression, a mean score of 7.74 ± 6.48 was obtained, and 107 participants (12.34%) reported a score of 0. On the CAS questionnaire, the mean score of the sample was 1.66 ± 2.82. However, 464 participants (52.6%) reported a score of 0, and when these participants were omitted from the analysis, the mean PHQ-9 score was 8.89 ± 6.20, and the mean CAS score was 3.48 ± 3.22.

When comparing the prevalence of substance use to the PQH9 and CAS scores, even though patients who consumed had higher scores on both scales, this was not statistically significant. Table 4 provides an exhaustive comparison. Participants who had a prior psychological diagnosis and received psychological therapy (weekly appointments, each lasting for approximately 1 h) showed a mean of 1.17 (±0.61) and 1.27 (±1) appointments before and during the pandemic, respectively (a 9% increase).

Assistance from religious or support groups to help deal with anxiety and depression did not significantly differ before (*p* = 0.580) and during (*p* = 0.605) the pandemic. On comparing the sample’s demographic characteristics and PHQ-9 and CAS scores, there were no significant differences between genders (*p* = 0.396 for the PHQ-9 score and *p* = 0.442 for the CAS score), having been infected and not being infected (*p* = 0.543 and *p* = 0.649), or having a COVID-19-infected acquaintance and not having such an acquaintance (*p* = 0.925 and *p* = 0.915).

A multiple linear regression analysis was computed to determine whether the PHQ-9 and CAS scales’ scores, age, and gender predict the use or not of any substance. The equation for the regression reported R2 = 0.077, indicating that just 7.7% of the variance in the number of cigarettes of tobacco by the PHQ-9 and CAS scales’ scores, age, and gender. The ANOVA equation showed to be significant F (4, 861) = 17.909, *p* = 0.001. Male participants were more likely to use any substance than female participants. Additionally, those participants with more age were more likely to use any substance than younger participants. Both gender and age were significant predictors of consumption. Also, a multiple linear regression was calculated if the PHQ-9 and CAS scales’ scores, age, and gender predict the number of cigarettes of tobacco smoked. The equation for the regression reported R2 = 0.016, indicating that just 1.2% of the variance in the number of cigarettes of tobacco by the PHQ-9 and CAS scales’ scores, age, and gender. The ANOVA equation showed to be significant F (4, 861) = 3.545, *p* = 0.007. Males presented smoked 2.5 more cigarettes than female participants, finding that gender was a significant predictor of the number of cigarettes of tobacco smoked.

Regression analysis to compare if the PHQ-9 and CAS scales’ scores, age, and gender predict the number of glasses of alcohol drunk or cigarettes of marijuana smoked. ANOVA equations were no-significant (alcohol: F (4, 861) = 0.111, *p* = 0.979, marijuana: F (4, 861) = 1.937, *p* = 0.102.

### 3.3. Gender Differences

**Figure 2:**
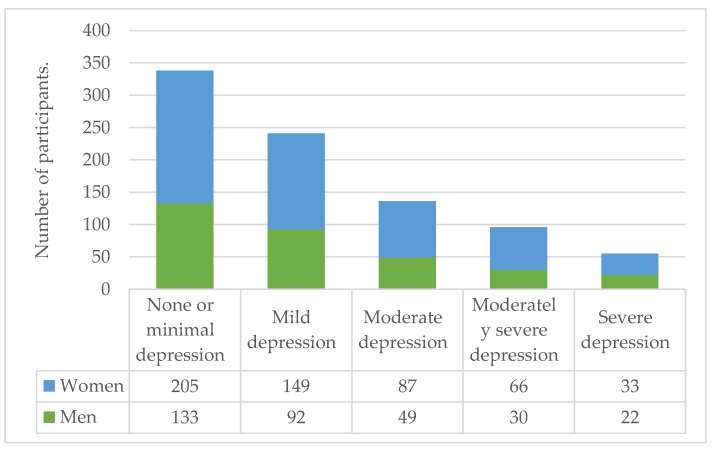
Classification groups based on the results of the PHQ-9 questionnaire.

**Figure 3:**
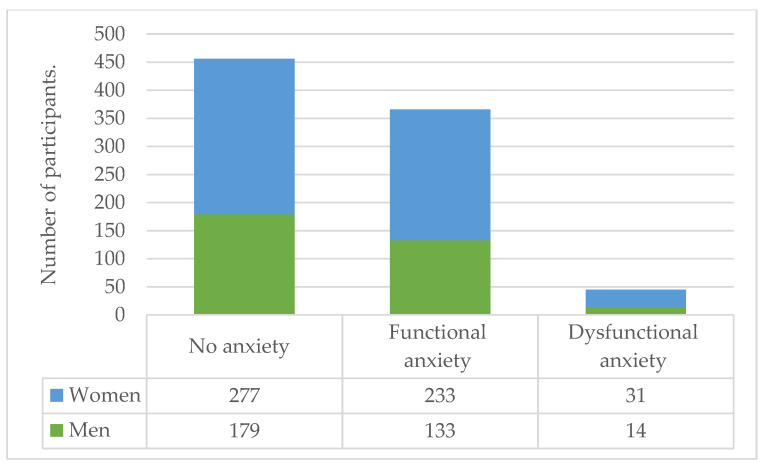
Classification groups based on the results of the CAS questionnaire.

When stratified between men and women, only alcohol consumption showed a statistically significant difference (*p* = 0.04); marijuana and tobacco consumption did not. Coffee consumption in women was markedly increased at 27.6% (*n* = 149) compared with that in men at 18.1% (*n* = 59). This difference was statistically significant (*p* = 0.002). Anxiety appeared to affect women at marginally higher rates than men, specifically functional (43% vs. 40.8%) and dysfunctional anxiety (5.7% vs. 4.3%). However, this difference was not significant (*p* = 0.08). The differences between men and women based on their substance consumption, PHQ-9 scores, CAS scores, and other responses to the pandemic are presented in Table 5, Figure 2 and Figure 3.

## 4. Discussion

The COVID-19 pandemic has led to social distancing, home isolation, and changes in habits and routines, which have abruptly modified the lifestyle of the entire population regardless of age, gender, and ethnicity. It has also increased psychosocial stress dependent on several factors, such as the decrease in economic, social, and educational activities, which is expected to lead to a deterioration of mental health associated with the improper intake and use of substances.

Substance use is defined as the consumption of a drug that can modify functions in an individual, altering mood, perception, reasoning, and conscience. The individual who consumes such substances considers these experiences relaxing, fun, and pleasant, and if the stimulation is constant, they will continue to consume them despite their consequences. That is why psychosocial stress, such as that caused by the pandemic, can predispose the population to start consuming substances for the first time or to increase their dose and frequency [23].

### 4.1. Substances Used

Substance use is correlated with stress. As stress becomes chronic, the vulnerability toward substance use or an increase in the dose of substances consumed increases. We report a substance consumption rate of 22.55% in our sample, which is almost double the incidence rate reported in 2019 by a Mexican national survey report (10.3%). Although the incidence rate was found to be much higher, we found a similar age distribution in both samples, with higher consumption rates in young male adults aged 18–34 years [24].

The distribution of substance use found in other studies tends to peak in adolescents and young adults, who tend to show more frequent and significant consumption of substances. This increased consumption of substances could be explained by social pressure and ideas, such as acceptable social behavior, despite the global efforts to reduce their use [25]. We found higher consumption of tobacco products in men than in women, which was similar to the findings of Marinho et al. [26], although their data were obtained from a sample aged >50 years, in contrast to our younger population.

One descriptive study with 4122 Mexican residents found a decrease in drug consumption, including tobacco, alcohol, and marijuana consumption, during the first months of the pandemic, which differs from the increased drug consumption reported in our study. This significant difference is likely related to the different time periods from which the data were collected. However, that study also reported increased tobacco consumption among patients presenting depressive symptomatology, which agrees with our findings [8].

### 4.2. Psychological Effects

In a study performed during the initial months of the COVID-19 pandemic in Hong Kong, Choi et al. [27] found that the pandemic had substantially impacted the population’s mental health. Of the 500 participants, 19% had depression (PHQ-9 score 310) and 14% had anxiety. In addition, 25% of the participants reported that their mental health had deteriorated since the beginning of the pandemic. A survey conducted in Japan revealed a correlation between feelings of isolation among workers during the pandemic and alcohol intake. Higher alcohol use related to increased feelings of loneliness [28] among patients who consumed alcohol more than once per week.

We found that 32% of the participants had PHQ-9 scores 310, which is considerably higher that the percentage reported by Choi et al. [27]. These different findings are again likely explained by the time periods in which both studies were conducted. Choi’s study was performed at the beginning of the pandemic when the population was starting to adapt to isolation and social distancing. In contrast, our study was performed 1 year after these measures were implemented worldwide, when the consequences of the present pandemic had already been observed on a larger scale. One cross-sectional survey of 9361 participants in Mexico reported higher levels of mental symptoms in individuals with full compliance with the lockdown procedures than in those with partial or no compliance [9].

Dubey et al. [29] analyzed the relationship between COVID-19 and addictions and found a considerable increase in addictive behaviors during the pandemic. This finding is perhaps explained by a notable delay in access to health services by people with addictive behaviors, encouraging this group of individuals to procure illegal drugs.

In Norway, a linear regression analysis of 4527 questionnaires revealed a correlation between depression, anticipation of financial loss due to the economic crisis brought on by the COVID-19 pandemic, and daily alcohol usage [30]. Alcohol, nicotine, and THC-containing products use increased by 25.8%, 28.8%, and 20.6%, respectively, during the pandemic, according to research carried out in Germany. Those who increased their substance use reported an increased incidence of anxiety and depression [31].

Differences between the genders distribution of substance consumption are known to be genetic and sociocultural. Historically, men tend to use and prefer substances categorized as recreational, and women tend to use prescribed substances [32]. This is consistent with our finding that men had a higher frequency of alcohol, tobacco, and marijuana consumption and higher rates of dose increase.

A comprehensive review of 45 studies on substance use during the pandemic revealed a consistent pattern of increased substance use, with the exception of alcohol, whose use reduced after the pandemic. Mental health factors were the most frequently mentioned in articles linking substance use [33]. A systematic review of alcohol, tobacco, cannabis, vaping, and other drug use among adolescents in April 2022 revealed a decrease in prevalence compared to previously recorded incidences [34].

Independent factors may account for the variation in outcomes among research. The cultural backdrop of each nation, its substance use pattern, the restrictions enforced during the pandemic, and the timespan during which the survey was conducted may influence the results. For this reason, it is crucial to continue future study to monitor this shifting scenario in populations that have received less attention, such as the Mexican [22,23].

Regarding the limitations of our study, the data were collected from a self-selected sample, which suggests a potential for bias in the characteristics of the participants. Due to the study’s retrospective design and the self-reporting nature of the data, the collection of data on drug use may have been biased. We aimed to circumvent the underreporting bias by employing a completely anonymous survey. Due to the anonymous nature of the survey, no methods were employed to eliminate possible duplicate responses. The present sample comprised users of legal substances and marijuana, with remaining substances underrepresented. Moreover, there was no reliable way to analyze the increment or decrement in medication since all participants used 32 different drugs, and no method was available to standardize the dosage.

## 5. Conclusions

Our participants mainly used alcohol and tobacco as the substances of choice to minimize the psychological effects of the COVID-19 pandemic. Before and during the pandemic, substance use increased considerably, and this increase was greater than the previous annual increase estimated from global observations. Furthermore, an increase in the dose of the preferred substance, particularly cigarettes and alcohol, showed a significant correlation with increased severity of de novo and prior psychological alterations. Therefore, consuming substances to reduce stress levels and depression led to a counterproductive effect, resulting in a synergic result. Since the present study was performed later in the pandemic, a high psychological impact was observed based on the PHQ-9 evaluation in the adult population studied in Mexico, revealing depression and stress directed toward COVID-19 in this population.

## Figures and Tables

**Table 1:** Demographic characteristics of the study sample.

Characteristics	Frequency	
Gender, *n* (%)		
Men	326 (37.6%)	
Women	540 (62.4%)	
Age, mean (standard deviation)	35.12 ± 13.31	
Educational degree, *n* (%)		
University degree	569 (65.7%)	
Master’s degree	92 (10.6%)	
Trade school	79 (9.1%)	
High school	75 (8.7%)	
Doctorate	18 (2.1%)	
Middle school	23 (2.7%)	
Post-doctorate	6 (0.7%)	
Primary school	4 (0.5%)	
COVID-19 infection, *n* (%)		
Yes	582 (66%)	
No	300 (34%)	
COVID-19-positive acquaintance, *n* (%)		
Yes	847 (97.5%)	
No	21 (2.4%)	
COVID-19 testing, *n* (%)		
Yes	524 (59.4%)	
No	358 (40.5%)	
Number of days between test and onset of symptoms		
<15 days	482 (92%)	
>15 days	42 (8%)	

COVID-19: coronavirus disease 2019.

**Table 2:** Comparison of substance consumption before and during the pandemic.

Substance	Number of Users before the Pandemic	Number of Users during the Pandemic	Percentage Change	*p* Value	df	*χ2*	Unit of Measurement	Mean Use/Dose before the Pandemic	Mean Use/Dose during the Pandemic	Percentage Change	*p* Value	df	*t* Score	Cohen’s *d*
Alcohol	165	172	4.24%	0.457	1	0.55	Glass (350 mL/day)	1.68 ± 1.63	2.06 ± 2.13	22.62%	0.006	136	−2.783	0.20
Tobacco	143	127	−11.19%	0.001	1	41.65	Cigarettes/day	15.85 ± 21.60	20.76 ± 29.85	30.98%	0.001	105	−3.889	0.18
Marijuana	49	50	2.04%	1.000	1	0.00	Cigarettes/week	2.80 ± 3.06	3.93 ± 6.32	40.36%	0.139	34	−1.514	0.38
OTC Drugs	Number of users before the pandemic	Number of users during the pandemic	Percentage change	*p* value	df	*χ2*	Unit of measurement	Mean use/dose before the pandemic	Mean use/dose during the pandemic	Percentage change	*p* value	df	*t* score	Cohen’s *d*
Alcohol	165	172	4.24%	0.457	1	0.55	Glass (350 mL/day)	1.68 ± 1.63	2.06 ± 2.13	22.62%	0.006	136	−2.783	0.20
Tobacco	143	127	−11.19%	0.001	1	41.65	Cigarettes/day	15.85 ± 21.60	20.76 ± 29.85	30.98%	0.001	105	−3.889	0.18
Marijuana	49	50	2.04%	1.000	1	0.00	Cigarettes/week	2.80 ± 3.06	3.93 ± 6.32	40.36%	0.139	34	−1.514	0.38

**Table 3:** Psychiatric pathologies and Patient Health Questionnaire-9 and Coronavirus Anxiety Scale mean scores of the sample population.

Previous Psychiatric Diagnosis, *n* (%)	*n* (%)
Anxiety	95 (10.96%)
Depression	76 (8.77%)
Empathy-associated burnout syndrome	2 (0.23%)
Panic disorder	4 (0.46%)
Psychotic disorder	1 (0.11%)
Obsessive-compulsive disorder	4 (0.46%)
Bipolar disorder	1 (0.11%)
PHQ-9	
Mean scores	7.74 ± 6.48
None or minimal depression	235 (27.1%)
Mild depression	245 (25.5%)
Moderate depression	111 (12.8%)
Moderately severe depression	96 (11%)
Severe depression	73 (8.4%)
CAS	
Mean score	1.66 ± 2.82
No anxiety	464 (52.6%)
Functional anxiety	373 (42.3%)
Dysfunctional anxiety	45 (5.1%)
Felt overwhelmed by the amount of information on COVID-19	
Yes	649 (74.7%)
No	219 (25.2%)
Felt prepared for the pandemic	
Yes	67 (7.7%)
No	801 (92.2%)

CAS, Coronavirus Anxiety Scale; COVID-19, coronavirus disease 2019; PHQ-9, Patient Health Questionnaire-9.

**Table 4:** Gender differences in substance use and psychological screening tests. Mean scores on the PHQ-9 and CAS by user group.

Substance	PHQ-9 Mean Scores	*p* Value	df	*t* Score	Cohen’s *d*	CAS Mean Scores	*p* Value	df	*t* Score	Cohen’s *d*
Any substance										
Yes	8.22 ± 6.61	0.236	864	−1.187	0.08	1.77 ± 2.87	0.445	864	−0.765	0.06
No	7.63 ± 6.45					1.60 ± 2.79				
Alcohol										
Yes	8.44 ± 6.80	0.140	864	0.589	0.12	1.88 ± 3.06	0.214	864	1.245	0.09
No	7.63 ± 6.41					1.59 ± 2.75				
Tobacco										
Yes	8.12 ± 6.47	0.644	864	0.462	0.05	1.66 ± 2.73	0.969	864	0.038	0.07
No	7.76 ± 6.50					1.64 ± 2.82				
Marijuana										
Yes	8.12 ± 5.87	0.710	864	−0.372	0.05	1.68 ± 2.30	0.938	864	−0.077	0.01
No	7.77 ± 6.53					1.64 ± 2.84				

Notes: *p*-values were obtained using the students’ *t* test. df: Degrees of freedom.

**Table 5:** Gender differences in substance use and psychological screening tests.

Alcohol Consumption	Men (*n* = 326)	Women (*n* = 540)	*p*-Value
Increase	40 (12.3%)	45 (8.3%)	0.040
No increase	286 (87.7%)	495 (91.7%)	
Marijuana consumption			
Increase	13 (4%)	18 (3.3%)	0.706
No increase	313 (96%)	522 (96.7%)	
Coffee consumption			
Increase	59 (18.1%)	149 (27.6%)	0.002
No increase	267 (81.9%)	391 (72.4%)	
Tobacco consumption			
Increase	34 (10.4%)	40 (7.4%)	0.133
No increase	292 (89.6%)	500 (92.6%)	
PHQ-9			
Mean scores	8.79 ± 6.20	8.94 ± 6.20	0.735
CAS			
Mean scores	3.44 ± 2.91	3.50 ± 3.39	0.865
Felt overwhelmed by amount of information on COVID-19			
Yes	201 (61.7%)	440 (81.5%)	0.001
No	125 (38.3%)	100 (18.5%)	
Felt prepared for the pandemic			
Yes	58 (17.8%)	21 (3.9%)	0.001
No	268 (82.2%)	519 (96.1%)	

## Data Availability

The datasets generated during and analyzed during the current study are available from the corresponding author upon reasonable request.
